# A Global Building Occupant Behavior Database

**DOI:** 10.1038/s41597-022-01475-3

**Published:** 2022-06-28

**Authors:** Bing Dong, Yapan Liu, Wei Mu, Zixin Jiang, Pratik Pandey, Tianzhen Hong, Bjarne Olesen, Thomas Lawrence, Zheng O’Neil, Clinton Andrews, Elie Azar, Karol Bandurski, Ronita Bardhan, Mateus Bavaresco, Christiane Berger, Jane Burry, Salvatore Carlucci, Karin Chvatal, Marilena De Simone, Silvia Erba, Nan Gao, Lindsay T. Graham, Camila Grassi, Rishee Jain, Sanjay Kumar, Mikkel Kjærgaard, Sepideh Korsavi, Jared Langevin, Zhengrong Li, Aleksandra Lipczynska, Ardeshir Mahdavi, Jeetika Malik, Max Marschall, Zoltan Nagy, Leticia Neves, William O’Brien, Song Pan, June Young Park, Ilaria Pigliautile, Cristina Piselli, Anna Laura Pisello, Hamed Nabizadeh Rafsanjani, Ricardo Forgiarini Rupp, Flora Salim, Stefano Schiavon, Jens Schwee, Andrew Sonta, Marianne Touchie, Andreas Wagner, Sinead Walsh, Zhe Wang, David M. Webber, Da Yan, Paolo Zangheri, Jingsi Zhang, Xiang Zhou, Xin Zhou

**Affiliations:** 1 Department of Mechanical and Aerospace Engineering, Syracuse University, 223 Link Hall, Syracuse, NY 13244 USA; 2 Building Technology and Urban Systems Division, Lawrence Berkeley National Laboratory, 1 Cyclotron Road, Berkeley, CA 94720 USA; 3 International Centre for Indoor Environment and Energy, Department of Civil Engineering, Technical University of Denmark, Kgs, Lyngby, Denmark; 4 106 Driftmier Engineering Center Annex, University of Georgia, Athens, GA 30602 USA; 5 Department of Mechanical Engineering, Texas A&M University, 400 Bizzell St, College Station, TX 77843 USA; 6 Edward J. Bloustein School of Planning & Public Policy, The State University of New Jersey, 33 Livingston Ave #383, New Brunswick, NJ 08901 USA; 7 Department of Industrial and Systems Engineering, Khalifa University of Science and Technology, PO Box 127788, Abu Dhabi, United Arab Emirates; 8 Department of Civil and Environmental Engineering, Carleton University, Ottawa, Canada; 9 Faculty of Environmental Engineering and Energy, Institute of Environmental Engineering and Building Installations, Poznań University of Technology, Berdychowo 4, 60-965 Poznań, Poland; 10 Centre for Urban Science and Engineering, Indian Institute of Technology Bombay, Mumbai, India; 11 Department of Architecture, University of Cambridge, CB2 1PX Cambridge, United Kingdom; 12 Laboratory of Energy Efficiency in Buildings, Department of Civil Engineering, Federal University of Santa Catarina, Florianópolis, Brazil; 13 Department of Architecture, Design and Media Technology, Aalborg University, Aalborg, Denmark; 14 School of Design, Swinburne University of Technology, Melbourne, Victoria 3122 Australia; 15 Energy, Environment and Water Research Center, The Cyprus Institute, Nicosia, Cyprus; 16 Institute of Architecture and Urbanism, University of São Paulo, Av. Trabalhador São-carlense, 400, São Carlos, SP CEP 13565-905 Brazil; 17 Department of Environmental Engineering (DIAm), University of Calabria, Rende, Italy; 18 eERG - end-use Efficiency Research Group, Department of Energy, Politecnico di Milano, Milan, Italy; 19 School of Computing Technologies, RMIT University, Melbourne, Victoria 3000 Australia; 20 Center for the Built Environment, University of California, Berkeley, USA; 21 Urban Informatics Lab, Department of Civil and Environmental Engineering, Stanford University, 473 Via Ortega Rm 269B, Stanford, CA 94305 USA; 22 Department of Mechanical Engineering, Dr B R Ambedkar National Institute of Technology, Jalandhar, Punjab 144011 India; 23 The Maersk Mc-Kinney Moller Institute, University of Southern Denmark, Odense, Denmark; 24 Department of Built Environment, University of Plymouth, Drake Circus, Plymouth, Devon, PL4 8AA UK; 25 Key Laboratory of Performance Evolution and Control for Engineering Structures, Ministry of Education, Tongji University, 1239 Siping Road, Shanghai, 200092 China; 26 Department of Heating, Ventilation and Dust Removal Technology, Faculty of Energy and Environmental Engineering, Silesian University of Technology, 44-100 Gliwice, Poland; 27 Berkeley Education Alliance for Research in Singapore Limited, Singapore, Singapore; 28 Department of Building Physics and Building Ecology, TU Wien, Vienna, Austria; 29 School of Architecture and Urban Design, RMIT University, Melbourne, Victoria 3000 Australia; 30 Intelligent Environments Laboratory, Department of Civil, Architectural and Environmental Engineering, The University of Texas at Austin, Austin, TX USA; 31 School of Civil Engineering, Architecture and Urban Design, University of Campinas, Campinas, Brazil; 32 Beijing Key Laboratory of Green Built Environment and Energy Efficient Technology, Beijing University of Technology, Beijing, 100124 China; 33 Department of Civil Engineering, The University of Texas at Arlington, Arlington, TX 76010 USA; 34 Department of Engineering, University of Perugia, Via G. Duranti 67, 06125 Perugia, Italy; 35 School of Engineering and Applied Sciences, Western Kentucky University, Bowling Green, KY 42101 USA; 36 School of Computer Science and Engineering, University of New South Wales (UNSW), Sydney, Australia; 37 Department of Civil and Mineral Engineering, Department of Mechanical and Industrial Engineering, University of Toronto, Toronto, Canada; 38 Karlsruhe Institute of Technology, Building Science Group, Karlsruhe, Germany; 39 Scanalytics Inc. N17W24222 Riverwood Dr. Suite 100, Waukesha, WI 53188 USA; 40 Building Energy Research Center, School of Architecture, Tsinghua University, Beijing, 100084 China; 41 Enea, Ispra, VA Italy; 42 School of Mechanical Engineering, Tongji University, Shanghai, 200092 China; 43 School of Architecture, Southeast University, #2 Si Pai Lou, Nanjing, 210096 China

**Keywords:** Energy and behaviour, Interdisciplinary studies

## Abstract

This paper introduces a database of 34 field-measured building occupant behavior datasets collected from 15 countries and 39 institutions across 10 climatic zones covering various building types in both commercial and residential sectors. This is a comprehensive global database about building occupant behavior. The database includes occupancy patterns (i.e., presence and people count) and occupant behaviors (i.e., interactions with devices, equipment, and technical systems in buildings). Brick schema models were developed to represent sensor and room metadata information. The database is publicly available, and a website was created for the public to access, query, and download specific datasets or the whole database interactively. The database can help to advance the knowledge and understanding of realistic occupancy patterns and human-building interactions with building systems (e.g., light switching, set-point changes on thermostats, fans on/off, etc.) and envelopes (e.g., window opening/closing). With these more realistic inputs of occupants’ schedules and their interactions with buildings and systems, building designers, energy modelers, and consultants can improve the accuracy of building energy simulation and building load forecasting.

## Abstract (summary)

**Table:** 

Measurement(s)	room temperature ambient air • room relative humidity • outdoor weather • window status • door status • fan status • HVAC measurement • lighting status • occupancy measurement • plug load • shade status
Technology Type(s)	Temperature Sensor Device • relative humidity sensor • weather station • window status sensor • door status sensor • fan status sensor • HVAC measurement sensors • lighting sensor • occupancy sensor • power sensor • shade status sensor

## Background & Summary

Commercial building, heating, ventilation, and air-conditioning (HVAC) systems account for nearly 10% of the global electric energy^1^. To enhance energy efficiency in the building sector, organizations such as the United States Green Building Council and the American Society of Heating, Refrigerating and Air Conditioning Engineers (ASHRAE), Federation of European Heating, Ventilation and Air Conditioning Associations (REHVA) or Chartered Institution of Building Services Engineers (CIBSE) have developed various design standards and guidelines (e.g. ASHRAE Standard 90.1 and 189.1, REHVA Guidebook 28^2^, 29^3^ and 31^4^, CIBSE Guideline F^5^ and L^6^) to support professionals in upgrading building design and construction practices. Furthermore, the bloom of building environmental assessment schemes and certifications – such as Building Research Establishment’s Environmental Assessment Method, Leadership in Energy and Environmental Design (LEED), the French Haute Qualité Environnementale, or the German Deutsche Gesellschaft für Nachhaltiges Bauen are promoting the construction and renovation of green and sustainable buildings, mostly in the developed countries. However, according to a recent study, LEED-certified buildings consume merely 10% less site energy than similar buildings that are not LEED-certified^7^. One of the major reasons for the underperformance of LEED-certified buildings is that operation of the building technical systems did not operate as intended, originating what is acknowledged as a performance gap motivating the need to explore the problem in more depth, using tools such as performance monitoring and benchmarking, information visualization, better controls, fault detection and diagnostics. But the limitation of these technologies in the building industry leads to a bottleneck in which no more energy savings can be achieved beyond those technologies. Estimating energy consumption in buildings to provide services is comprehensively influenced by architectural design, engineering technologies and cultural background, operational practices, occupant behavior, social equity, etc. Among them, occupant behavior has been suggested to be one of the most important factors influencing that explain the performance gap in buildings^8^.

However, the challenges in studying occupant behavior in buildings are many including: (a) Occupant behavior is dynamic and complex in nature; (b) Privacy issues make data collection difficult; and (c) Monitoring occupant behavior relies on various types of sensors with relatively high costs. To better understand the occupant behavior in buildings, more than 500 papers have been published on the topic of occupant behavior over the last decade^9–12^. Examples of these studies include: (1) Occupant presence; (2) Occupant number; (3) Opening/closing windows; (4) Opening/closing window blinds; (5) Turning on/off lights; (6) Adjusting thermostats; (7) Turning on/off air-conditioners; (8) HVAC sizing and thermal comfort; (9) Crowd control and security; and (10) Circulation design^9^. Models from these studies were built to describe occupant behavior in buildings in order to evaluate the performance of building design and operation^9^. Each research study has its own dataset and represents an individual case, making cumulative learning a key challenge although the studies were carried out around the world. With such a large body of data to work on, occupant behavior researchers will not be able to dive deeper to compare occupant behaviors across various building types and nations, or derive valuable information for energy-efficient building design and operations based on limited field-measured data.

**Fig. 1:**
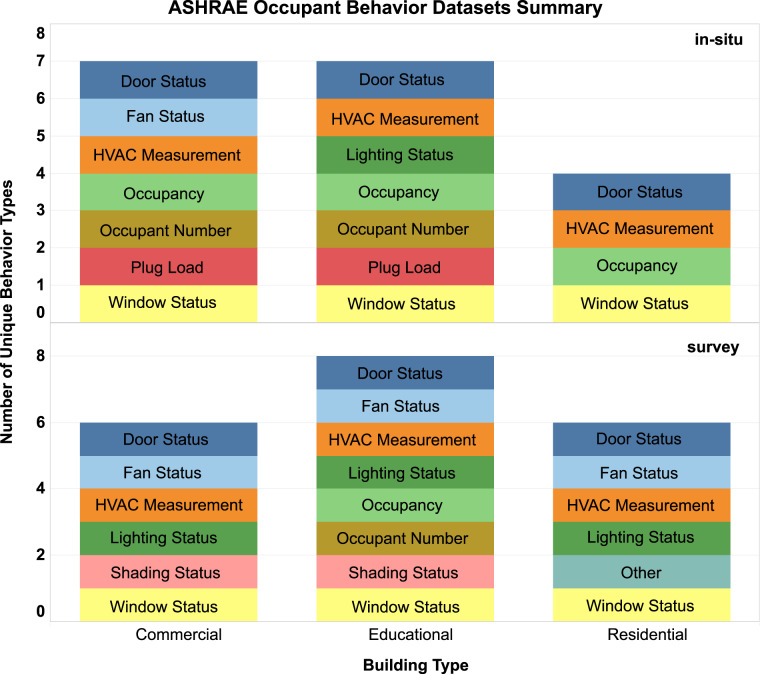
Summary of the ASHRAE Global Occupant Behavior Database by building types.

In this paper, we develop and present the worldwide ASHRAE occupant behavior database (https://ashraeobdatabase.com) with data contributions from researchers across the globe, as part of the IEA EBC Annex 79 project^10^. A prior effort, under the IEA EBC Annex 66 project^11^, has published five occupant behavior datasets, which were also included in this database^13^. The database consists of 34 datasets from 39 institutions located in 15 countries and 10 climate zones. This is a comprehensive global occupant behavior database. The Brick schema^12^ was adopted to develop sensor and room metadata models. As shown in Figure 1, the database includes 11 different types of occupant behavior measurements collected from 3 different types of building spaces. The database can support various use cases of occupant behavior research, including:

- Understand occupant behaviors in real buildings,
- Compare and understand the diversity and dynamics of occupant behaviors,
- Develop mathematical models of occupant behaviors at various spatial and temporal resolutions by building types,
- Benchmark various occupant behavior modeling approaches,
- Generate typical occupant schedules and behavior models for use in building performance simulations, as well as building energy codes and standards.

## Methods

**Fig. 2:**
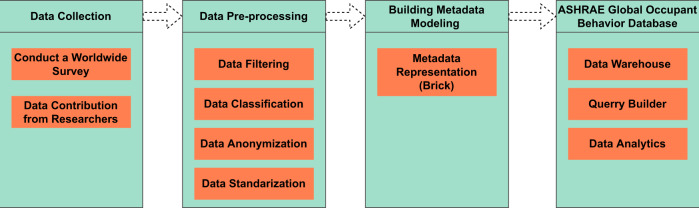
Overview of the technical approach.

This section introduces the data collection, pre-processing and modeling approaches implemented in this study. Figure 2 shows the detailed technical approach we have followed to develop the occupant behavior database.

### Data collection

Before collecting the data, a worldwide survey was developed and administered among researchers willing to contribute their datasets. As Table 1 shows, the survey contains some basic questions about the metadata, type of occupant behavior, data collection method, period and frequency, geographic location and building type, heating/cooling strategy (interaction between occupant and thermostat), climate zone. With the information collected from the worldwide survey, the project team reached out to potential contributors with detailed requirements. Below is a list of preliminary data requirements:

- Data should come from field experiments, and represent “real” occupant behavior in real buildings;
- The time span of the dataset should be at least one month to represent weekly and monthly behavior patterns, or represent any behavior changes within buildings;
- Data on adaptive behavior (e.g., opening or closing windows to maintain thermal comfort) should come with indoor and outdoor environmental parameters (e.g., ambient and indoor air temperature);
- The dataset should contain metadata information, a dictionary of data headings, experimental setup details, and data collection methods.

**Table 1:** Dataset description in the survey (*Required field).

**A. Building Information**
A1. Building Location*
(City, State/Province, Country)
A2. Building Type*
(Commercial, Educational, Residential)
A3. Building Function*
(University, Office, Apartment….)
A4. Climate Zone
A4. Year Built
A5. Number of floors
**B. Zone Information**
B1. Space Type*
(Office, Bedroom, Conference Room…)
B2. Area(if not in m2, please specify)
B3. Zone Drawing
B4. Window Orientation (North, West, SW….)
B5. Window Operation Type (Manual, Automatic…)
B6. Shading Device (if applicable)
**C. Building Equipment Information**
C1.Cooling Info(if applicable)
C1-1. Cooling type
C1-2. Control Type (Remote, Thermostat…)
C2. Heating Info(if applicable)
C2-1. Heating Type
C2-2. Control Type (Remote, Thermostat…)
C3. Hot Water Info(if applicable)
C3-1. Hot Water Heating Type
**D. Data Collection Information**
D1. Occupant Behavior Sensor Info (If applicable)
D1-1. Sensor(s) Type
D1-2. Variable Measured
D1-3. Collection Interval
D1-4. Sensor Location
D1-5. Range
D1-6. Accuracy
D. Data Collection Information
D2. Indoor Environment Sensor Info (If applicable)
D2-1. Sensor(s) Type
D2-2. Variable Measured
D2-3. Collection Interval
D2-4. Sensor Location
D2-5. Range
D2-6. Accuracy
D3. Outdoor Sensor Info (If applicable)
D3-1. Sensor(s) Type
D3-2. Variable Measured
D3-3. Collection Interval
D3-4. Sensor Location
D3-5. Range (if not in SI Unit, please specify)
D3-6. Accuracy
(if not in SI Unit, please specify)
D4. Weather Station Info(if applicable)
D4-1. Weather Station Distance
D4-2. Variable Measured
D4-3. Collection Interval
D5. Survey Collection Info(if applicable)
D5-1. Survey Type(Observer, self-report…)
D5-2. Variable Measured
D5-3. Collection Interval
**E. Dataset Information**
E1. Occupant Behavior Info
E1-1. Occupant behavior studied
E2. Collection Period
E2-1. Start Time* (YYYY-MM-DD)
E2-1. End Time*(YYYY-MM-DD)
E2-3. Missing Dates
E3. Description of each folder (if applicable)
E4. Description of Data Files by Column
(If not in SI unit, please specify)
**F. Additional Information**

### Data Pre-processing

After receiving raw datasets from contributors, each dataset was inspected based on the above requirements. The contributors were responsible for addressing privacy that relevant for occupant data^14^ and further anonymization was added as part of pre-processing. All datasets were then separated into survey-based, *in-situ*-based, and mixed-type of data. The *in-situ*-based data contains dynamic information and measurements in the building with constant sampling intervals, such as door and window status (OPEN/CLOSED), indoor equipment status (ON/OFF), indoor environment information (temperature, humidity, CO_2_ concentration, illumination, etc.). Survey-based data contains information from the specific study, including occupant questionnaires, static information about the building envelope, floor plan and sampled measurement. Datasets without a continuous and fixed sampling time interval were also classified as survey-based data. One dataset was categorized as mixed type data since it has both survey-based and *in-situ*-based data. Table 2 provides a review of all the datasets, including the country of origin, collection method, and measurement categories. There are in total 24 *in-situ*-based datasets, one mixed-type of dataset, and nine survey-based datasets.

**Table 2:** Summary of 34 datasets.

Dataset ID	Country	Building Types	Dataset Types & Publications	Door Status (ON/OF)	Fan Status (ON/OFF)	Window Status (ON/OFF)	Shade Status (ON/OFF)	Occupant Number	Lighting Status (ON/OFF)	Occupant Presence	Plug Load	HVAC Measurements	Indoor Measurements	Outdoor Measurements	Others
1	UK	E	survey^13,20^	X	X	X	X	X		X			X	X	
2	USA	C	*in-situ*^21^								X				
3	India	R	survey^22,23^	X	X	X							X	X	
4	Denmark	E	*in-situ*^24^					X				X	X		
5	Italy	E	*in-situ*^25^	X		X					X		X	X	
6	Brazil	E	*in-situ*			X			X			X	X	X	
7	Australia	E	*in-situ*^26^							X		X	X	X	
8	Canada	R	*in-situ*^27^	X		X							X	X	
9	Canada	E	*in-situ*^28^						X	X					
10	Italy	E	*in-situ*^29^	X		X		X		X	X	X	X		
11	USA	R	*in-situ*^30^							X					
12	China	E	survey			X	X					X	X	X	
13	Poland	R	*in-situ*^31^									X		X	
14	China	E	*in-situ*			X				X			X	X	
15	China	C, E, R	*in-situ*	X		X						X	X	X	
16	Brazil	C	*in-situ*			X						X	X	X	
17	China	E	*in-situ*	X				X				X	X	X	
18	UAE	E	*in-situ*^32^							X	X		X		
19	Singapore	C	survey^33^		X	X						X	X	X	
20	Austria	E	*in-situ*^34^								X		X	X	
21	China	C	*in-situ*			X				X		X	X	X	
22	USA	E	*in-situ*^35^								X				
23	Brazil	C	*in-situ*^36^			X						X	X		
24	Germany	C	*in-situ*^37^			X				X			X		
25	Brazil	C	*in-situ*^38^		X	X						X	X	X	
26	USA	C	mixed^39^	X	X	X	X	X				X	X	X	
27	USA	R	survey^40^												X
28	USA	R	survey^40^												X
29	USA	R	survey^40^												X
30	USA	E	*in-situ*^41^						X	X			X		
31	India	R	survey^42^	X	X	X			X			X	X	X	
32	USA	C	*in-situ*^43^					X					X		
33	USA	C	*in-situ*					X							
34	Italy	E	survey	X	X	X	X		X			X	X	X	

(Building Types: C – Commercial; E – Educational; R – Residential).

Table 2 listed the types of occupant behavior data that were included in the database. Each type of measurement has a CSV template file associated with it. Based on the templates, all the raw data were pre-processed to be consistent in standard naming, data types, and formats. The data types follow the entities and tags defined in the Brick schema, which is covered in the following section.

The detailed data pre-processing procedure includes the following:

- Empty columns from the raw datasets were removed;
- All the missing values in raw datasets were replaced with - 999;
- As a process of anonymization, each building and room were assigned with a unique ID number;
- The headings of common data columns in the raw data were standardized following pre-defined dataset templates. Survey data with unique questions or measurement naming remained the same as the original naming schema. For the survey-based data, a dictionary of headings was created for each dataset;
- The format of timestamp in the raw data was revised to follow the format “yyyy-mm-dd hh:mm:ss”, the time zone remained as local time zone to reflect the daily behavior patterns of the occupant; The time granularity of some datasets is at the minute level. In that case, the value of seconds in the timestamp was kept as zero.
- The decimal point of the raw data was adjusted accordingly, such as status (binary), occupant number data (integers), data of indoor and outdoor conditions (one decimal precision digit);
- Raw occupancy data that only contains enter/leave events were aggregated to get a total number of occupants in the space.

### Sensor and room metadata modeling

Contextual information provided by subsystem vendors as “metadata”, that is, the data about data, those subsystems include HVAC systems, lighting, security, and sensing/monitoring systems^15^. In this database, each dataset contains one or more buildings with various types of sensors installed to measure occupant behavior patterns, indoor and outdoor conditions as shown in Table 2. The Brick schema was adopted to develop sensor and room metadata models, to better present the information of different types of measurements and relationships between subsystems with buildings. Brick is an open-source unified metadata schema for buildings, which standardizes semantic descriptions of the assets and their relationships in buildings. The assets include physical, logical, and virtual assets. The core concepts of Brick are Tag, Class, Relationship, and Graph. A Tag represents an atomic fact or attribute of an entity. A Class is a category with a definition used to represent a group of entities in the building. The Relationship defines the nature of the link between two related entities. A graph is a summarized figure indicating the data structure of a set of entities and their relationships. Brick defines a detailed ontology (https://brickschema.org/ontology) to support and expand these core concepts. The database users can easily extract sensor and room metadata information (e.g., number of rooms in the building, number and types of sensors that were deployed in the space) without querying the database.

### ASHRAE global occupant behavior database

A website (https://ashraeobdatabase.com) was created as a data warehouse for public access. Query builder tools were developed based on different behavior types, cities and countries, building types, study ID, and publication list. Users can select and download data from the database interactively with the query tools. Data analytic functions were developed to provide an interactive overview of the database and assist users to select the dataset.

## Data Records

**Fig. 3:**
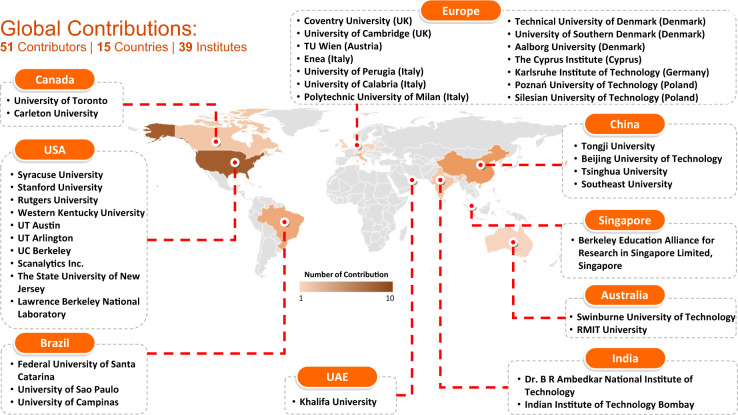
Global Contributions to the ASHRAE Occupant Behavior Database.

Figure 3 shows the geographical and institutional details of the global contributions to this occupant behavior database. Köppen-Geiger climate classification has been widely used in the smart building area by researchers around the world^16–18^. Since the datasets^19^ were contributed by researchers around the globe, Köppen-Geiger climate classification was adopted to represent the different climate zones in the datasets. The database covers 10 different climate zones globally according to the Köppen-Geiger climate classification (https://en.climate-data.org), which covers Tropical rainforest, Tropical savanna wet, Hot deserts, Humid subtropical, Temperate oceanic, Hot-summer Mediterranean, Cool-summer Mediterranean, Hot-summer humid continental, Warm-summer humid continental, Monsoon-influenced hot-summer humid continental climates. All datasets were compressed into a zip file named “ASHRAE Global Occupant Behavior Database”, with a total size of 548 MB. The final datasets have been uploaded to the figshare website for public use^19^. A website (https://ashraeobdatabase.com) was created to query and download the desired data from the database based on different selection criteria.

**Fig. 4:**
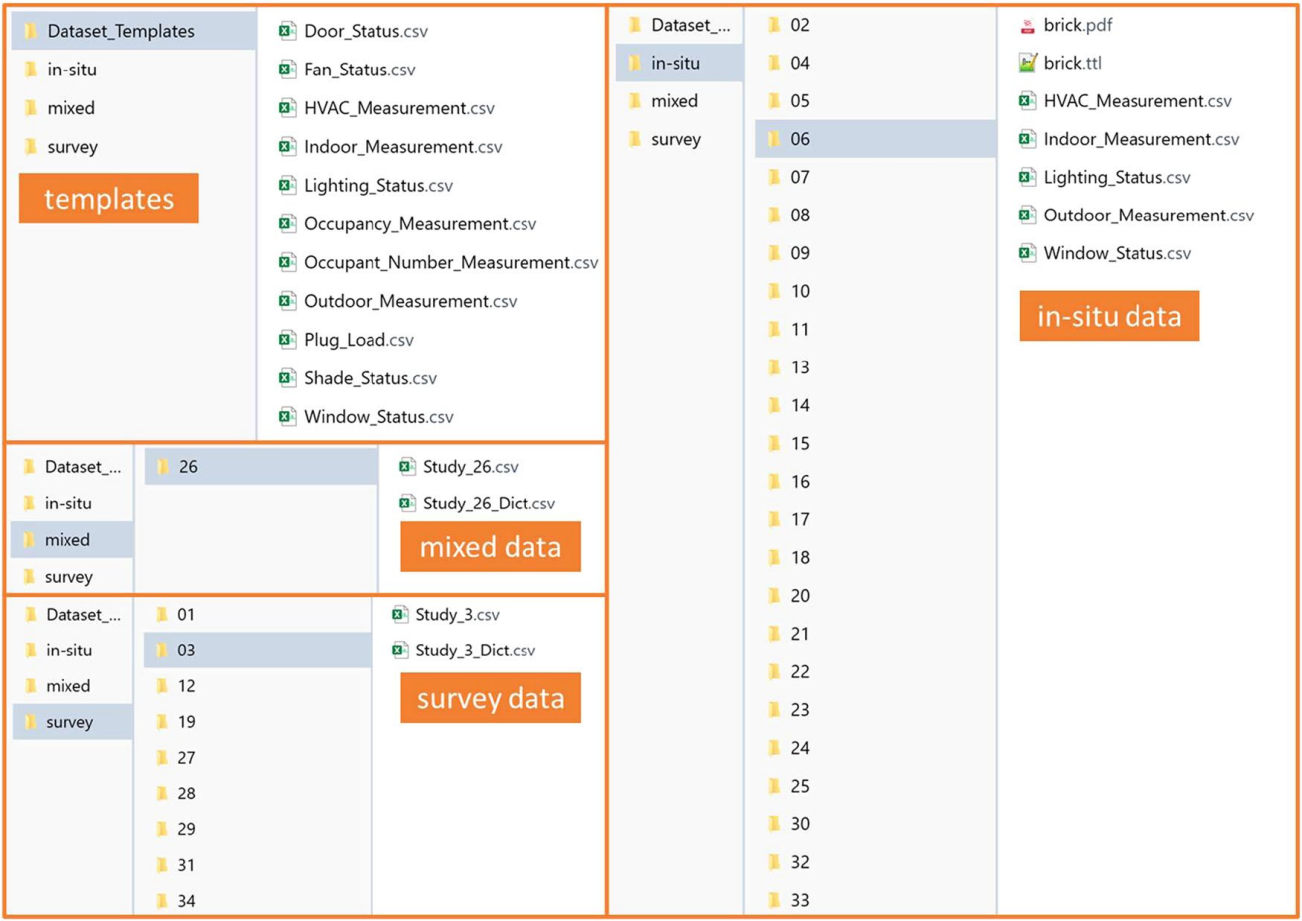
Folder view of the database.

As Figure 4 indicates, in the root folder, a folder named “Dataset_Templates” contains all the templates (.csv files) that have been used to process raw datasets. Those templates can be used as references for future data contributors. The “*in-situ*” folder contains 22 datasets representing the different dynamic measurement data (.csv files) with constant sampling intervals. The folder also includes the brick.pdf file which is a PDF view of the Brick model, and the brick.ttl (Turtle) file, which is the Brick model that can be viewed interactively through the Brick server (https://viewer.brickschema.org/). In the PDF file, users can get a glimpse of sensor measurement types and relationships with the building. Through the Turtle file, users can extract the complete sensor and room metadata information of the dataset without opening those datasets. The “survey” folder contains questionnaires as well as dynamic or static measurement data without a constant sample interval time. Survey-based data varies greatly, as different research projects focus on various measurements and questions. Each dataset has a dictionary of headings to assist users with understanding the data. In total, 12 survey-based datasets were collected in this database.

### Sensor and room metadata

**Fig. 5:**
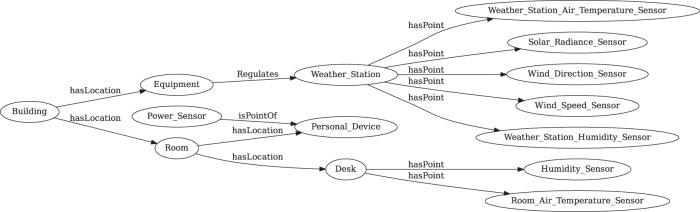
View of the Brick model of Dataset 20.

As discussed above, 22 Brick models have been developed for the *in-situ* datasets. Figure 5 shows an example of the Brick model for Dataset 20. The data contributor collected plug load, indoor and outdoor measurements from an educational office building in Vienna, Austria. Data collection started on January 1, 2013 and ended on December 31, 2013. The building has six different rooms, plug load data was collected from four out of six rooms, and indoor measurements were collected from all the rooms. This Brick model covers all the entities and their relationships in this dataset. The relationship Room “isLocationOf” Desk indicates that desk-level measurements exist in this dataset. In this model, there can be multiple “Building”, “Room”, and “Desk”. The number of those entities can be extracted from the Turtle file. The relationships include “isLocationOf”, “hasPoint”, and “Regulates”. Those relationships could be reversed as “hasLocation”, “isPointOf”, and “isRegulatedBy”. The points represent different sensors, for instance, wind direction sensor, wind speed sensor, air temperature sensor, humidity sensor, etc. Detailed information such as numbers or names of the entities and points can be found in the Turtle file.

## Technical Validation

In this section, we explore the datasets and perform an initial analysis of different occupant behavior data. The analysis focuses on occupant number, door opening, occupant presence, window opening, and outdoor measurements.

### Occupant number historical data

**Fig. 6:**
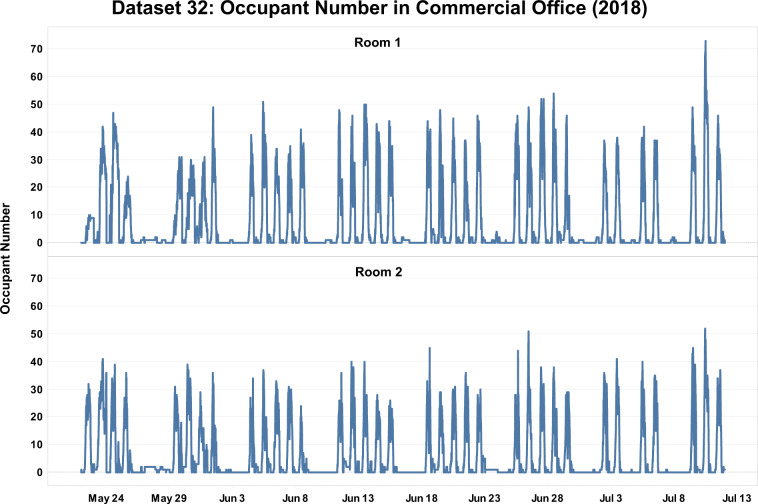
Historical occupant number data from Dataset 32.

**Fig. 7:**
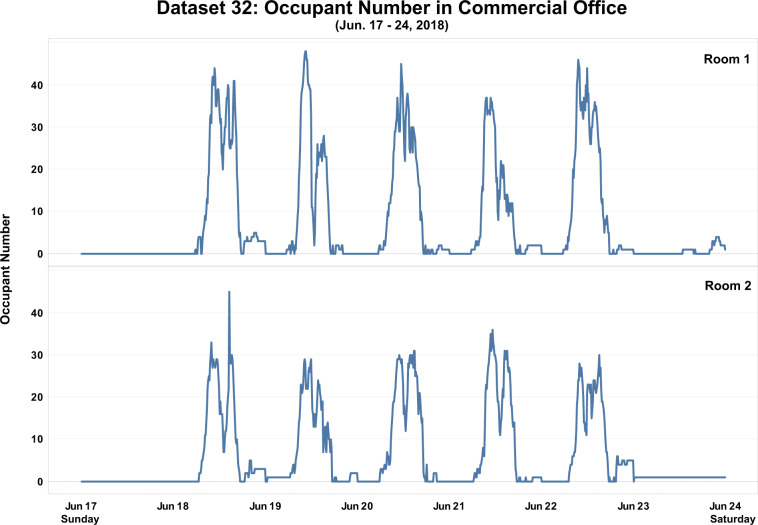
Historical occupant number data from Dataset 32 in one week.

Figure 6 shows the historical data of occupant number in a commercial office building from Dataset 32. The occupancy of two office rooms was measured from May 22, 2018 to July 11, 2018. Camera-based sensors were deployed to collect occupant counts in this study. To valid the dataset, researchers added an automatic daily calibration for the measurement, which includes two functions: first, set the occupant count to zero if it is less than zero; second, set the occupant count to zero at 3 AM each day. From the figure, we can observe the weekday and weekend trends of occupant number in both rooms. Holiday effects can also be captured, such as Memorial Day (Monday - May 28, 2018), and Independence Day (Wednesday - July 4, 2018). The occupant number dropped to relatively lower values during weekends and holidays. Figure 7 provides a detailed view of the historical occupant number in one week, a common workday schedule was observed from both rooms.

### Door status

**Fig. 8:**
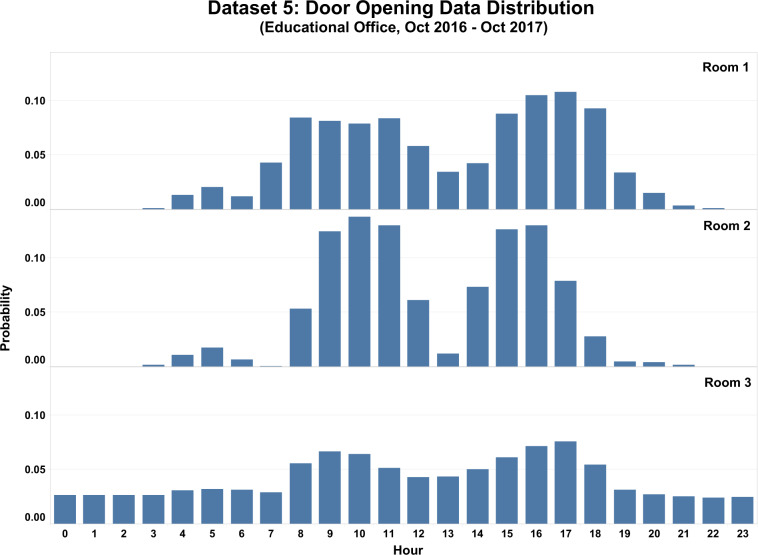
Data distribution of door openings from Dataset 5.

Figure 8 shows a data distribution of door status (Open/Closed) in educational offices from Dataset 5. The building in this dataset has multiple rooms, with each room having only one door. Cable-connected magnetic sensors were used to measure the opening/closing of doors in this study. The data was collected from October 27, 2016, to October 31, 2017, with 5-minute granularity. The figure indicates that Room 1 and 2 have a similar trend of the door opening activities during the working hours (8 AM–5 PM). However, Room 3 showed different trends where door opening behaviors spread across the 24 hours of a day. Overall, the door opening probability was low, which indicated that the doors remained closed most of the time.

### Occupant presence

**Fig. 9:**
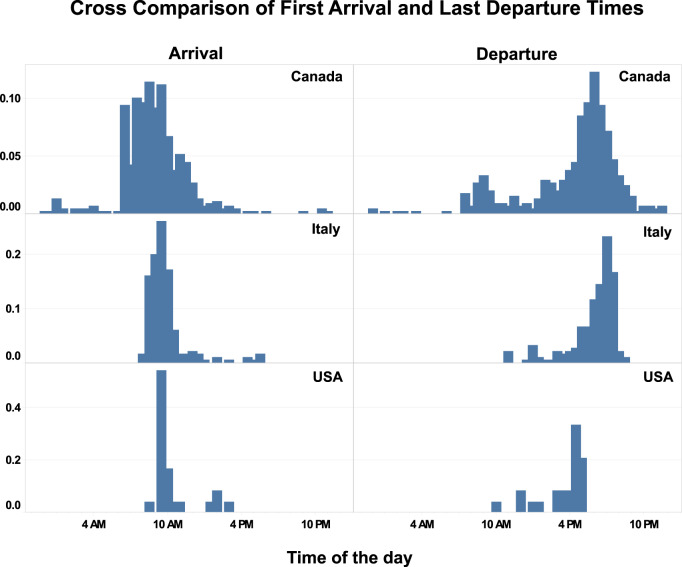
Cross-comparison of first arrival and last departure between occupant presence datasets from different countries.

Figure 9 shows a cross-comparison of the first arrival and last departure times among three different datasets (Dataset 9, 10 and 30) from three different countries. The data was collected from educational offices in various months from 2016 to 2018. Passive infrared sensors were used in Dataset 9 to collect the event-based occupancy data, the minimum occupied time was set as 15 minutes. Dataset 10 monitored space occupancy manually by a person in the office. And Dataset 30 utilized Bluetooth device pairing technology to sample occupancy data every one minute. In Figure 9, the first arrival captures the time when the space is first occupied, while the last departure captures the time when the space is last occupied during the day. Even though the distribution varies because of different lengths of data collection periods, it is clear that first arrival times are centered around 10 AM during the day. And the last departure times during the day are centered between 6 PM and 7 PM.

### Window operations

**Fig. 10:**
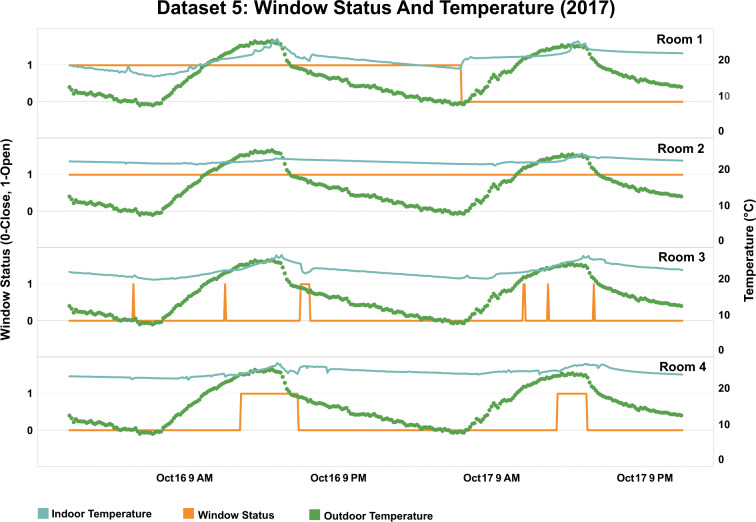
Window operation coupled with indoor and outdoor temperature in Dataset 5.

Figure 10 shows window status data with indoor and outdoor temperature measurements from Dataset 5. The data was collected from educational offices. Researchers deployed cable-connected magnetic sensors to measure the opening/closing of windows in this study, the sampling time was five minutes. The indoor temperature was measured by the temperature probe (PT1000 class A Cable) with an accuracy of 0.15 °C or less at 0 °C. Outdoor temperature was collected every 10 minutes from an over roof weather station that was installed about 10 meters above the ground. From the changes in indoor and outdoor temperature over two days, it can be observed that the HVAC system helped to maintain an indoor temperature close to 24 °C during the day. In Rooms 3 and 4, window opening activities were captured during the afternoons on both days when the indoor and outdoor temperature both were relatively high.

### Outdoor measurements

**Fig. 11:**
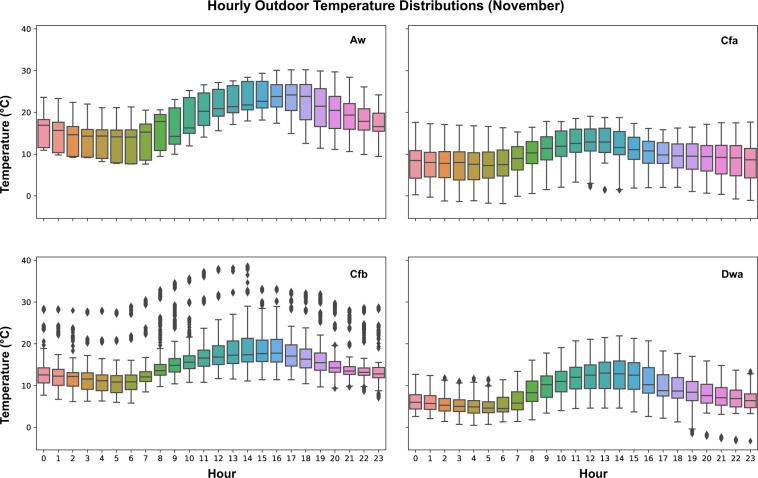
Outdoor temperature distributions by hour in different datasets and climate zones. Dataset 16 – Aw; Dataset 5 – Cfa; Dataset 7- Cfb; Dataset 14 – Dwa.

**Fig. 12:**
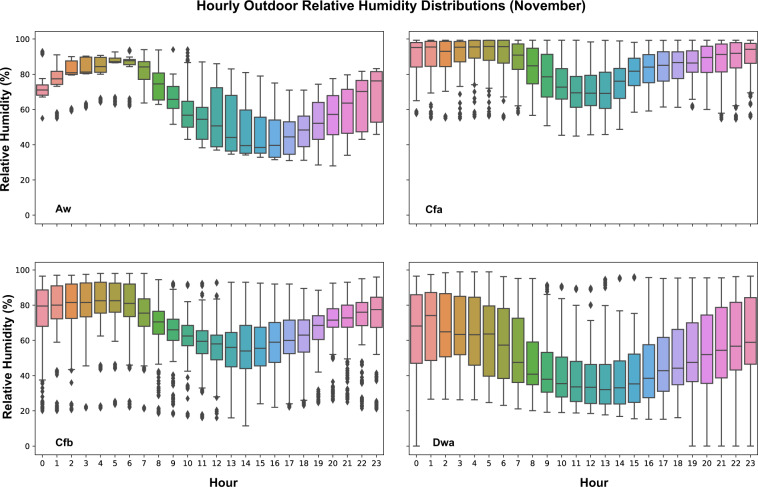
Outdoor relative humidity distributions by hour in different datasets and climate zones. Dataset 16 – Aw; Dataset 5 – Cfa; Dataset 7- Cfb; Dataset 14 – Dwa.

**Fig. 13:**
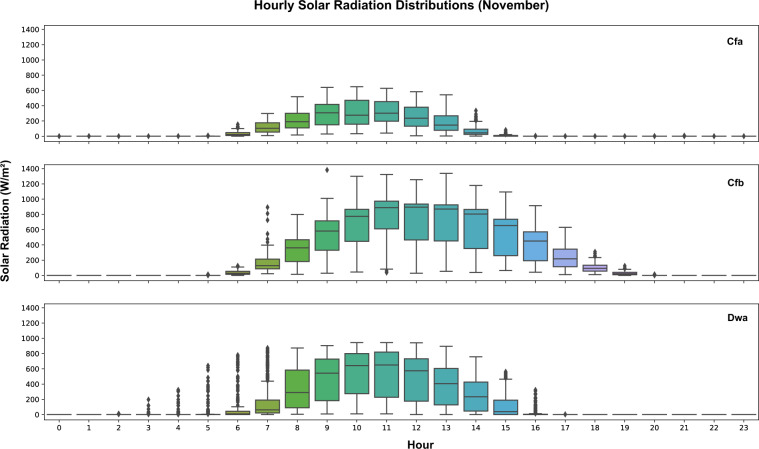
Outdoor solar radiation distributions by hour in different datasets and climate zones. Dataset 5 – Cfa; Dataset 7- Cfb; Dataset 14 – Dwa.

Field measurements of outdoor parameters were investigated based on the available datasets. Those outdoor measurements cover five different climate zones, such as Tropical savanna wet climate (Aw), Humid subtropical climate (Cfa), Temperate oceanic climate (Cfb), Monsoon-influenced hot-summer humid continental climate (Dwa), and Warm-summer humid continental climate (Dfb). The time granularity varies among those datasets. Dataset 5 collected outdoor measurements from an over roof weather station with a 10-minute sampling time. Dataset 7 has a 5-minute sampling interval and data was collected from an onsite outdoor weather station. Researchers of Dataset 14 installed over roof portable outdoor weather stations and sampled outdoor measurements every 10 minutes. However, Dataset 16 collected hourly outdoor measurements from local weather station which is approximately 4 kilometers away from the most experimental buildings. Then, the data was resampled into every 10 minutes using linear interpolation. Since datasets were collected from different months in various years, in order to compare outdoor measurements in the same time span, data from November of four datasets (5, 7, 14, and 16) were identified and analyzed. Hourly data from different days were analyzed and plotted using boxplot. Figure 11 shows hourly outdoor temperature distributions of four different datasets and climate zones. Figure 12 shows hourly outdoor relative humidity distributions of those datasets. Figure 13 shows the hourly outdoor solar radiation distributions in datasets 5, 7, and 14 since dataset 16 doesn’t measure outdoor solar radiation. The results captured different trends of temperature, relative humidity, and solar radiation within the four different climate zones by the time of the day.

## Usage Notes

The datasets^19^ have been uploaded to a public domain of the figshare website, users can download data through this link (10.6084/m9.figshare.16920118.v6). A website (https://ashraeobdatabase.com) was created to query and download the desired data from the database based on different selection criteria. These criteria include types of measurement data, countries and cities, type of building, study ID, and publication. The website also provides an overall analysis of all the datasets, a list of available publications from those studies, etc.

## Data Availability

All the codes used to clean the raw datasets have been uploaded to GitHub for public use (https://github.com/yapanliu/ashrae-ob-database). The raw datasets are also open to the public on request.
